# Non-Reconciled Physical-Layer Keys-Assisted Secure Communication Scheme Based on Channel Correlation

**DOI:** 10.3390/e24081167

**Published:** 2022-08-22

**Authors:** Meng Wang, Kaizhi Huang, Zheng Wan, Xiaoli Sun, Liang Jin, Kai Zhao

**Affiliations:** 1 School of Cyber Science and Engineering, Zhengzhou University, Zhengzhou 450001, China; 2 Institute of Information Technology, PLA Strategic Support Force Information Engineering University, Zhengzhou 450001, China; 3 Purple Mountain Laboratories, Nanjing 211111, China

**Keywords:** physical-layer security, key generation, channel correlation, information reconciliation

## Abstract

Physical-layer key generation technology requires information reconciliation to correct channel estimation errors between two legitimate users. However, sending the reconciliation signals over the public channel increases the communication overhead and the risk of information leakage. Aiming at the problem, integrated secure communication schemes using non-reconciled keys have attracted extensive attention. These schemes exploit channel coding to correct both inconsistent keys and transmission error bits. Meanwhile, more redundant code bits must be added to correct errors, which results in a lower secure transmission rate. To address the problem, we analyze the merit of channel correlation between non-reconciled key generation and secure transmission. Inspired by this, we propose a non-reconciled physical-layer keys-assisted secure communication scheme based on channel correlation. First of all, the signal frame is designed to make use of channel correlation between non-reconciled key generation and secure transmission. Based on the channel correlation, non-reconciled keys are then generated from the wireless channel to encrypt transmitted data. Moreover, an adaptive coding algorithm based on the equivalent channel is presented to encode the data bits before encryption, to guarantee reliable transmission. Finally, theoretical analysis and simulations demonstrate the significant performance of the proposed scheme in terms of low bit error ratio and high secure transmission rate.

## 1. Introduction

With the continuous evolution of wireless communication technology, ubiquitous mobile communication has become an indispensable part of our daily life. In particular, fifth-generation (5G) wireless communications transmit a large amount of information with high data rates and extremely low latency, which further promotes the rapid development of the Internet of Things (IoT) [1,2,3,4]. Meanwhile, information security is always the main concern in wireless communications because the open and broadcast nature of the wireless medium leads to inherent vulnerability to eavesdropping attacks. Thus, considerable research focuses on classic public-key cryptography techniques to ensure the confidentiality of transmitted information [5]. However, public-key infrastructure is required to be established in these techniques, which leads to difficulties in key distribution and management in resource-limited communication systems. In addition, these techniques rely on the computational complexity of mathematical problems and are thus at risk of being broken by powerful quantum computers in the foreseeable future [6].

Recently, the physical-layer key generation technique, which is considered a supplement to upper-layer public key cryptography techniques, has attracted widespread attention [7,8]. As a secure approach to key distribution and management based on the wireless channel, this technique can exploit the characteristics of the wireless channel to generate random keys from the wireless channel. Specifically, based on the reciprocity and temporal variation of the wireless channel, two legitimate users can extract unpredictable and highly correlated channel state information (CSI) after probing channels. Moreover, the spatial decorrelation of the wireless channel prevents eavesdroppers from obtaining the relevant information of the legitimate channels, to ensure the security of keys generated by legitimate users [9]. The physical-layer key generation consists of four steps, i.e., channel estimation, quantization, information reconciliation, and privacy amplification [10]. In general, the channel estimates of two legitimate users are inconsistent due to channel fading and noise. Thus, to generate identical symmetric keys, information reconciliation is required by sending signals to correct channel estimation errors between two legitimate users. There are several common information reconciliation methods such as cascade [11] and BBBSS [12]. In order to improve reconciliation efficiency, the authors in [13] designed a new hybrid information reconciliation protocol. The reconciled keys are used to encrypt transmitted data information to achieve secure transmission, which can be termed a reconciled physical-layer keys-based secure communication (RK-SC) scheme [14]. However, the reconciliation signals are sent over the public channel, which increases the communication overhead and the risk of eavesdropping attacks.

Therefore, increasing attention is being directed at integrated schemes such as physical-layer secure communication schemes using non-reconciled keys. Note that an integrated non-reconciled physical-layer keys-based secure communication (NRK-SC) scheme was first proposed in [15]. This scheme generates keys without information reconciliation but shares the error correction capability of channel coding. Based on [15], the authors in [16] designed an efficient polar code that could significantly improve the secure communication efficiency of the NRK-SC scheme. Moreover, the authors in [17] evaluated the superior performance of the NRK-SC scheme compared to the RK-SC scheme by deriving the bit error ratio (BER), channel capacity, and security capacity. Similarly, it was proved that the NRK-SC scheme outperformed the RK-SC scheme in terms of communication overhead, computation complexity, and the secure transmission rate in [18]. However, note that the existing NRK-SC scheme no longer corrects inconsistent keys via information reconciliation but instead adds key errors to the transmission process. Thus, channel coding with a stronger ability is required to correct both key errors and transmitted error bits, which leads to a lower secure transmission rate. This continues to be a significant problem for the existing NRK-SC scheme.

Aiming at this problem, this paper conducts a comprehensive and theoretical study on non-reconciled key generation and secure transmission. Furthermore, the potential merit of channel correlation between key generation and secure transmission is analyzed. Motivated by this advantage, we present a non-reconciled physical-layer keys-assisted secure communication scheme based on channel correlation. The main contributions are as follows:

- We design a signal frame to adjust the pilot and data signals transmission process, which contributes to utilizing channel correlation between non-reconciled key generation and secure transmission.
- Based on the channel correlation, the non-reconciled keys generated from the wireless channel are used to encrypt transmitted data. Before encryption, we propose an adaptive coding algorithm based on the equivalent channel to encode data bits according to different signal-to-noise ratios (SNRs).
- Theoretical analysis and simulation results demonstrate that compared with the NRK-SC scheme, the proposed scheme has significant effects in reducing BER and improving the secure transmission rate.

The rest of this paper is organized as follows. Section 2 deals with the system model and introduces the problem statement with respect to the prior scheme. The non-reconciled physical-layer keys-assisted secure communication scheme based on channel correlation is proposed in Section 3. The security and reliability of the proposed scheme are evaluated in Section 4 through mathematical analysis. Section 5 presents the simulation results, and Section 6 concludes the paper.

## 2. System Model and Problem Statement

### 2.1. System Model

**Figure 1:**
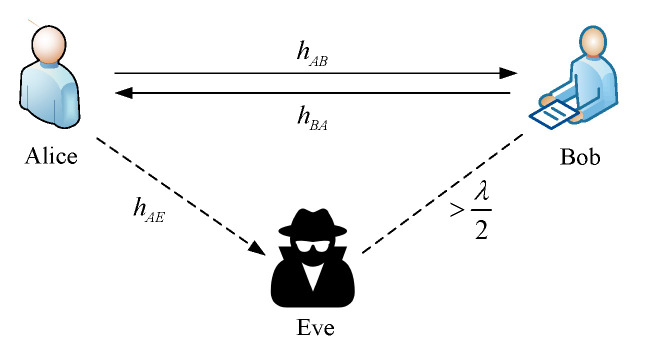
System model.

We consider a single-input single-output single-eavesdropper model that operates in time-division duplex (TDD) mode, as shown in Figure 1. All the users are equipped with a single antenna. Two legitimate users, Alice and Bob, wish to establish shared keys to ensure secure communication in the presence of the passive eavesdropper, Eve. The wireless channel is a slowly varying Rayleigh fading channel and remains constant during the coherence time, where $h_{AB},h_{BA}$∼CN(0,1). We define $h_{AB}$, $h_{BA}$, and $h_{AE}$ as denoting the channels from Alice to Bob, Bob to Alice, and Alice to Eve, respectively. According to the reciprocal of the wireless channel, $h_{AB}=h_{BA}$. Eve is located more than half a wavelength away from legitimate users. In addition, Eve and the legitimate users have no line of sight (LoS). Therefore, Eve experiences a wireless channel independent of that of the legitimate users, because they are spatially decorrelated between different geographic locations [19,20]. We assume that Alice and Bob have the same transmitting power, i.e., $P_{A}=P_{B}=P$, where the power allocation parameters of the pilot and data signals are α and 1−α, respectively. In addition, Eve knows the complete communication process and the information transmitted over public channels. Alice and Bob probe the channel to generate keys, which are employed to encrypt data information to realize secure communication.

### 2.2. Problem Statement

**Figure 2:**
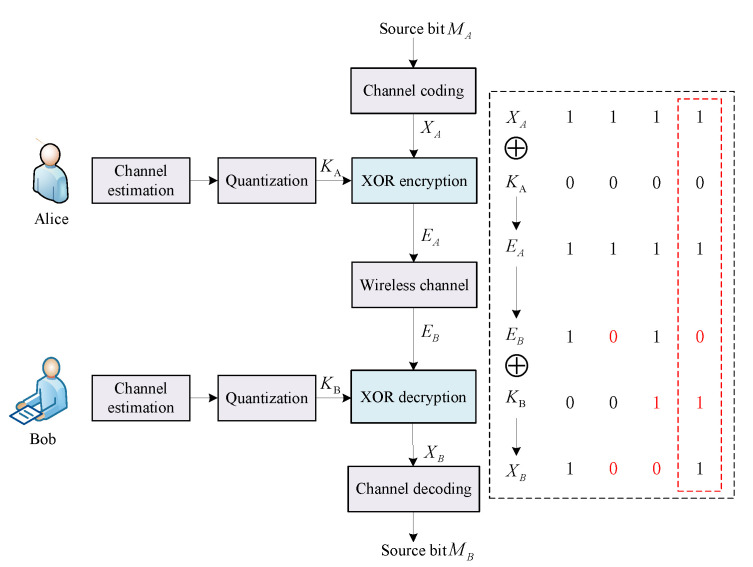
The existing NRK-SC scheme.

As mentioned in the Introduction, the NRK-SC scheme [15,16,17,18] realizes secure communication using non-reconciled keys to deal with information leakage and the communication overhead caused by information reconciliation. For comparison purposes, we provide a brief description of the existing NRK-SC scheme [15,16,17,18]. As shown in Figure 2, the non-reconciled keys $K_{A}$ and $K_{B}$ are generated by Alice and Bob via channel estimation and quantization. Note that since the inconsistent keys are not corrected via information reconciliation, channel coding is required to correct not only transmitted error bits but also inconsistent keys. After that, the generated keys $K_{A}$ are used to encrypt encoded source bits $X_{A}$ via exclusive-OR (XOR) encryption. Next, the ciphertexts $E_{A}$ are transmitted to Bob over the wireless channel. Then, Bob receives the ciphertexts $E_{B}$ and recovers $X_{B}$ from the ciphertexts via XOR decryption. The source bits $M_{B}$ can be obtained after channel decoding.

In the XOR operation process, when the key and transmitted bit errors occur simultaneously, the errors can be corrected. As shown in Figure 2, note that $E_{B}$ and $K_{B}$ errors occur. The decrypted bit received by Bob $X_{B}=E_{B}⊕K_{B}$ is correct. Thus, when there exists a channel correlation between key generation and secure transmission, the error probabilities of keys and transmitted bits are correlated, which can result in the achievement of an excellent error correction effect via the XOR operation. This is the potential advantage that channel correlation can offer.

However, in the existing NRK-SC scheme, key generation and secure transmission are performed at different coherence times. The keys generated by the previous coherence time are used to encrypt the encoded data bits of the next coherence time. Thus, the error probability of keys is different from that of the transmitted bits, and the advantage does not apply. Moreover, non-reconciled keys share the error correction capability of channel coding in the NRK-SC scheme. Thus, more redundant code bits must be added to correct error bits, leading to a lower coding efficiency and a lower secure transmission rate. Therefore, to address the problem and fully use the advantage of channel correlation, we propose a non-reconciled physical-layer keys-assisted secure communication scheme based on channel correlation.

## 3. The Proposed Non-Reconciled Keys-Assisted Secure Communication Scheme Based on Channel Correlation

**Figure 3:**
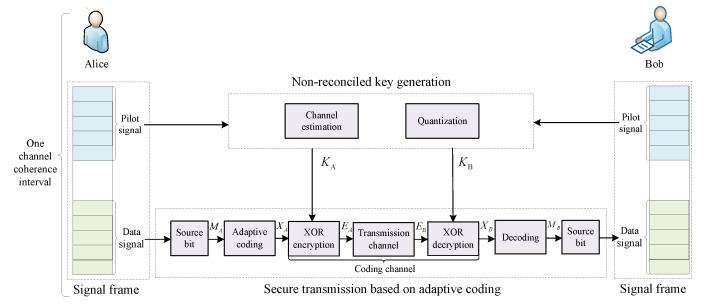
The proposed non-reconciled keys-assisted secure communication scheme based on channel correlation.

As illustrated in Figure 3, the proposed scheme has three steps, namely, signal frame design, non-reconciled key generation, and secure transmission based on adaptive coding. The first step is aimed at designing the signal transmission process to fully use channel correlation between key generation and secure transmission. In the second step, the wireless channel is probed to generate non-reconciled keys. Subsequently, in the third step, the generated keys are used to encrypt encoded data bits to achieve secure transmission, where data source bits are encoded by the adaptive coding based on the equivalent channel. In the following, we discuss the three steps in detail.

### 3.1. Signal Frame Design

**Figure 4:**
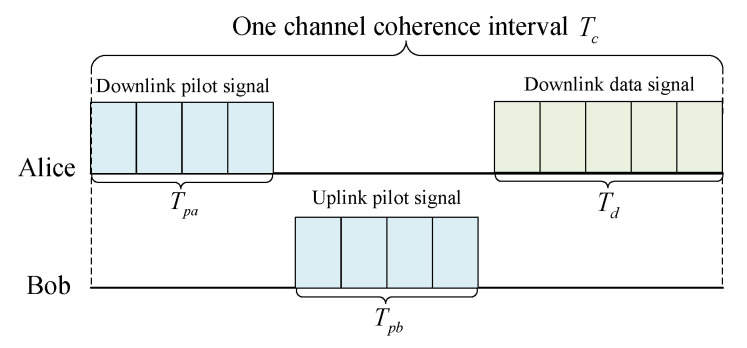
Signal frame design of downlink commmunication.

To take advantage of channel correlation between non-reconciled key generation and secure transmission, we design the signal frame to perform these two processes at one channel coherence time. As shown in Figure 4, we take downlink commmunication as an example. The downlink and uplink pilot lengths are $T_{pa}$ and $T_{pb}$, respectively, where $T_{pa}=T_{pb}$. The downlink data length is $T_{d}$. In addition, $T_{c}$ represents the interval length of one channel coherence time. Firstly, two legitimate users send downlink and uplink pilot signals for channel probing to generate the keys. Subsequently, we transmit data signals after the pilot signals. The keys are used to encrypt the encoded data source bits. In addition, the length of the transmitted signals must satisfy

(1) $$T_{pa}+T_{pb}+T_{d}<T_{c}.$$

The design of short-packet communication with limited packet length can meet the low-latency requirements of wireless communication. It is especially suitable for real-time communication scenarios. In addition, we consider that the signal power ratio is equal to the length ratio, i.e., $\alpha =\frac{T_{pa}}{T_{pa}+T_{d}}$. Hence, we can minimize the BER of the communication system by reasonably setting the power allocation parameters α, which will be further introduced in Section 5.

### 3.2. Non-Reconciled Physical-Layer Key Generation

There are two mainstream approaches for legitimate users to generate keys from the wireless channel in TDD wireless communication systems. One involves estimating the CSI using the received pilot signals, and the other involves sending random signals in order to mix the random signals and the channel gains [21]. The former is selected in our scheme.

During the key generation process, Alice and Bob first send pilot signals to each other to extract the CSI from the wireless channel. The transmitting pilot power values of Alice and Bob are $\sigma_{A}^{2}$ and $\sigma_{B}^{2}$, respectively. The channel noise is additive white Gaussian noise (AWGN) with zero mean and variance $\sigma_{n}^{2}$. Hereafter, Alice, Bob, and Eve use the least-squares (LS) algorithm to estimate the channel. The channel estimates can be expressed as

(2) $$\left\{\begin{array}{c} {\hat{h}}_{AB}=h_{AB}+w_{A}\\ {\hat{h}}_{BA}=h_{BA}+w_{B}\\ {\hat{h}}_{AE}=h_{AE}+w_{E} \end{array}\right.$$

where $w_{A}$, $w_{B}$, and $w_{E}$ denote channel estimation errors. They are independent identically distributed complex Gaussian random variables, with zero mean and variance $\sigma_{n}^{2}/\sigma^{2}$, i.e., $w_{B}$ and $w_{E}$ are $CN\left(0,\sigma_{n}^{2}/\sigma_{A}^{2}\right)$ and $w_{A}$∼$CN\left(0,\sigma_{n}^{2}/\sigma_{B}^{2}\right)$.

Then, Alice and Bob quantize the channel estimates ${\hat{h}}_{AB}$ and ${\hat{h}}_{BA}$. In order to make the distribution of the quantized sequences in each quantization interval as uniform as possible, we consider using an equal probability quantization algorithm to quantize the channel estimates [17]. The probability distribution function (PDF) of the channel estimates is used to divide the quantization interval. To extract more keys, we quantize the real part and the imaginary part of the channel estimates. The quantized sequences are converted into Gray code with length *q*, where *q* represents the quantization precision. After that, the non-reconciled keys $K_{A}$ and $K_{B}$ can be obtained.

### 3.3. Secure Transmission Based on Adaptive Coding

When using the coding method with a low code rate to encode transmitted data bits at high SNR, more redundant code bits must be added to correct errors, resulting in lower coding efficiency. However, at low SNR, using a high code rate cannot fully correct inconsistent keys and transmitted error bits, making it difficult to ensure reliable communication. Thus, to maximize the secure transmission rate at different SNRs, an adaptive coding algorithm using BCH code (n,k,t) based on the equivalent channel is presented. We use BCH code in this paper since it is widely used due to its lightweight and low-complexity advantages. An equivalent channel that contains an encryption channel and a transmission channel is constructed to compute the BER. Next, the calculation results are used to further design the optimal parameters of *n* and *k*. Finally, according to *n* and *k*, we can encode data bits to guarantee reliable transmission at the maximum secure transmission rate. The adaptive coding algorithm based on the equivalent channel is summarized in Algorithm 1.
**Algorithm 1** Adaptive coding algorithm based on the equivalent channel**Input:** Source bits $M_{A}$; code length *n*, and SNR $\gamma_{p}$, $\gamma_{d}$**Output:** Encoded bits $X_{A}$1:Calculate the upper bound of the BER based on the equivalent channel $P_{et}^{UB}=P_{e}+P_{AB}^{\mathrm{key}}-2P_{e}P_{AB}^{\mathrm{key}}$.2:Maximize secure transmission rate $R^{UB}=\frac{n-2nP_{et}^{UB}-1}{n}$.3:Calculate information bits $k^{UB}=nR^{UB}=n-2nP_{et}^{UB}-1$.4:Fix the code length *n* and adjust $k^{UB}$ according to the BER and encode source bits $M_{A}$ to obtain encoded bits $X_{A}$ by using *n* and $k^{UB}$.5:**Return**$X_{A}$.

#### 3.3.1. Adaptive Coding Based on the Equivalent Channel

We define the channel between adaptive coding and decoding as the coding channel, as indicated in Figure 3. To facilitate analysis, an equivalent channel is set up to calculate the BER of the coding channel in step 1 of Algorithm 1. We will introduce the model in detail in the following.

**Figure 5:**
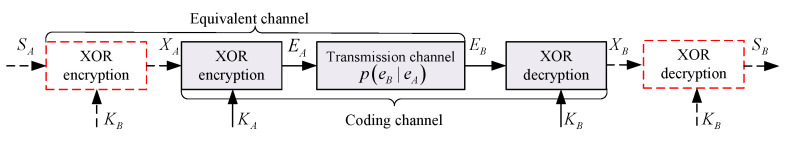
Equivalent channel model.

As shown in Figure 5, the coding channel is composed of the encryption channel, transmission channel, and decryption channel, where $K_{A}$ and $K_{B}$ represent keys for performing encryption and decryption, respectively. The keys $K_{A}$ and $K_{B}$ are not exactly identical. Note that a pair of symmetrical virtual encryption and decryption modules are added at both ends of the coding channel in order to build the equivalent channel. The virtual XOR encryption and decryption use symmetric keys $K_{B}$. We assume that the input and output alphabets of virtual encryption and decryption modules are $S_{A}$ and $S_{B}$, respectively, and that source bits are binary encoded. Therefore, the coding channel is a binary discrete memoryless channel (DMC) with a probability space of $\left[X_{A},p\left(x_{B}|x_{A}\right),X_{B}\right]$, where $p\left(x_{B}|x_{A}\right)$ denotes the transfer probability of the coding channel. Here, $x_{A}$ and $x_{B}$ are symbols taken from $X_{A}$ and $X_{B}$, respectively, where $x_{A},x_{B}\in \left\{0,1\right\}$. The transmission channel formed by the modulator, wireless channel, and demodulator is also a DMC, which can be expressed as $\left[E_{A},p\left(e_{B}|e_{A}\right),E_{B}\right]$, where $p\left(e_{B}|e_{A}\right)$ denotes the transfer probability of the transmission channel, while $e_{A}\in E_{A}$ denote ciphertexts which are encrypted by keys $K_{A}$ and $e_{B}\in E_{B}$ denote ciphertexts recovered by the demodulator. The equivalent channel consists of an encryption channel $\left[S_{A},p\left(e_{A}|s_{A}\right),E_{A}\right]$ and a transmission channel $\left[E_{A},p\left(e_{B}|e_{A}\right),E_{B}\right]$ cascade.

Note that there is no new noise or interference added at the virtual XOR encryption and decryption modules. Thus, the transfer probability of the coding channel does not change after adding the virtual modules, which can be shown as $p\left(s_{B}|s_{A}\right)=p\left(x_{B}|x_{A}\right)$, where $s_{A}\in S_{A}$, $s_{B}\in S_{B}$. In addition, according to the principles of the binary field, we have $S_{B}=E_{B}$. This means that $p\left(s_{B}|s_{A}\right)=p\left(e_{B}|s_{A}\right)$. Therefore, we can obtain

(3) $$p\left(s_{B}|s_{A}\right)=p\left(e_{B}|s_{A}\right)=p\left(x_{B}|x_{A}\right).$$

In other words, we can derive the BER of the coding channel from the equivalent channel, which can be computed as

(4) $$\begin{array}{cc} P_{et}=p\left(e_{B}|s_{A}\right) & \overset{\left(a\right)}{=}\sum_{e_{A}\in E_{A}}p\left(e_{B}|e_{A}s_{A}\right)p\left(e_{A}|s_{A}\right)\\ \; & \overset{\left(b\right)}{\leq }\sum_{e_{A}\in E_{A}}p\left(e_{B}|e_{A}\right)p\left(e_{A}|s_{A}\right)\\ \; & =P_{e}+P_{AB}^{\mathrm{key}}-2P_{e}P_{AB}^{\mathrm{key}} \end{array}$$

where (a) is given by the total probability formula and (b) represents $p\left(e_{B}|e_{A}s_{A}\right)\leq p\left(e_{B}|e_{A}\right)$, since the encryption channel and the transmission channel are correlated such that $p\left(e_{B}|e_{A}s_{A}\right)$$=p\left(e_{B}|e_{A}\right)$ holds when $E_{B}$ only depends on $E_{A}$ and is independent of $S_{A}$. In addition, $P_{AB}^{\mathrm{key}}$ and $P_{e}$ represent the key disagreement rate (KDR) and the transmission bit error rate (TBER), respectively. Therefore, the upper bound of the BER of the coding channel is $P_{et}^{UB}=P_{e}+P_{AB}^{\mathrm{key}}-2P_{e}P_{AB}^{\mathrm{key}}$.

Next, to calculate the BER of the coding channel, we solve the KDR and the TBER according to the SNR. Taking the real part of the channel estimates as an example, the 1-bit quantized KDR of ${\hat{h}}_{BA}$ and ${\hat{h}}_{AB}$ can be derived as

(5) PABkey=Pr[Re(h^BA)>0,Re(h^AB)<0]+Pr[Re(h^BA)<0,Re(h^AB)>0]=2×∫−∞∞Pr[Re(h^BA)>0,Re(h^AB)<0|Re(hAB)=u]fRe(hAB)(u)du=12−1π∫0∞erf2u2αPσn2exp−u2du.

According to [17], The TBER of the BPSK signal transmitted in the Rayleigh fading channel can be calculated as

(6) $$P_{e}=\frac{1}{2}\left(1-\sqrt{\frac{\gamma_{d}}{1+\gamma_{d}}}\right)=\frac{1}{2}\left(1-\sqrt{\frac{P(1-\alpha )}{P\left(1-\alpha \right)+\sigma_{n}^{2}}}\right).$$

We take $\gamma_{p}=\frac{\alpha P}{\sigma_{n}^{2}}$ and $\gamma_{d}=\frac{P(1-\alpha )}{\sigma_{n}^{2}}$ to represent the SNRs of the pilot signals of Alice or Bob and the SNR of the received data signals, respectively.

In step 2, to maximize the secure transmission rate, the problem can be expressed as

(7) $$\begin{array}{cc} \max R & =\frac{k}{n}\\ s.t.\frac{t}{n} & \geq P_{et}\\ n-k & \geq 2t+1 \end{array}$$

To solve this problem, we can compute that

(8) $$t\geq nP_{et}$$

(9) k≤n−(2t+1)

In this way, we can derive that $k\leq n-2nP_{et}-1$. Hence, the upper bound of the secure transmission rate is

(10) $$R^{UB}=\frac{k^{UB}}{n}=\frac{n-2nP_{et}^{UB}-1}{n}.$$

Furthermore, $k^{UB}=nR^{UB}=n-2nP_{et}^{UB}-1$ can be calculated in step 3. By exploiting the relationship between *n* and $k^{UB}$, we can obtain the maximum value of the optimal parameter *k* by fixing *n*. According to *n* and the maximum value of *k*, we can use BCH code (n,k,t) to encode source bits $M_{A}$ to obtain $X_{A}$ in step 4.

#### 3.3.2. Secure Transmission

The encoded bits $X_{A}$ are encrypted by keys $K_{A}$, and we can obtain $E_{A}=X_{A}⊕K_{A}$. Then, the ciphertexts $E_{A}$ are transmitted through the transmission channel, and Bob receives the ciphertexts $E_{B}$. The source bits recovered by Bob can be expressed as

(11) $$\begin{array}{cc} M_{B} & =\mathrm{Decode}(E_{B}⊕K_{B})\\ \; & =\mathrm{Decode}(\mathrm{Encode}\left(M_{A}\right)⊕K_{A}⊕K_{B})\\ \; & =\mathrm{Decode}(\mathrm{Encode}\left(M_{A}\right)⊕\varepsilon ) \end{array}$$

where $\varepsilon =K_{A}⊕K_{B}$ represents the difference between $K_{A}$ and $K_{B}$. Note that there is a difference between $E_{A}$ and $E_{B}$ due to channel fading and noise, which can be corrected by adaptive coding.

## 4. Performance Evaluation

In this section, the performance of the proposed scheme is evaluated using security analysis and reliability analysis.

### 4.1. Security Analysis

Since the eavesdropping channel and the legitimate channels are independent of each other, we consider that the encryption channel and the transmission channel are also independent for Eve. Similarly to (4), The BER of Eve can be calculated as

(12) $$P_{Eve}=P_{e}+P_{AE}^{\mathrm{key}}-2P_{AE}^{\mathrm{key}}P_{e}$$

where the KDR of Alice and Eve is given by

(13) PAEkey=Pr[Re(h^AB)>0,Re(h^AE)<0]+Pr[Re(h^AB)<0,Re(h^AE)>0]=2×Pr[Re(h^AB)>0]Pr[Re(h^AE)<0]=12

where Equation (13) holds due to the independence of the legitimate channels and the eavesdropping channel. Thus, we substitute $P_{AE}^{\mathrm{key}}=\frac{1}{2}$ into (12) to obtain $P_{Eve}=\frac{1}{2}$, which indicates that Eve cannot steal any key information via the eavesdropping channel. The security of the communication system is guaranteed.

### 4.2. Reliability Analysis

From (4), we have $P_{et}\leq P_{e}+P_{AB}^{\mathrm{key}}-2P_{e}P_{AB}^{\mathrm{key}}$, where $P_{eN}=P_{e}+P_{AB}^{\mathrm{key}}-2P_{e}P_{AB}^{\mathrm{key}}$ represents the BER of the existing NRK-SC scheme [15,16,17,18]. Note that the proposed scheme has a lower BER compared to the NRK-SC scheme. This is due to the fact that the errors are corrected via an XOR operation when the encryption channel $\left[S_{A},p\left(e_{A}|s_{A}\right),E_{A}\right]$ and the transmission channel $\left[E_{A},p\left(e_{B}|e_{A}\right),E_{B}\right]$ are correlated. From (4) and (10), the comparison of the secure transmission rate can be expressed as

(14) $$R_{I}^{UB}\geq R_{N}^{UB}$$

where $R_{N}^{UB}$ and $R_{I}^{UB}$ represent the upper bounds of the secure transmission rate of the NRK-SC scheme and our proposed scheme, respectively. It is worth noting that our proposed scheme has superior performance compared to the NRK-SC scheme with respect to the secure transmission rate.

Moreover, we analyze the reliability of the proposed scheme. When $X_{A}$ obeys an equal probability distribution, i.e., $\mathrm{Pr}\left[x_{A}=1\right]=\mathrm{Pr}\left[x_{A}=0\right]=0.5$, the channel capacity can be derived as

(15) $$\begin{array}{cc} C & =1-H(p\left(x_{B}|x_{A}\right))\\ \; & \geq 1-H(P_{e}+P_{AB}^{\mathrm{key}}-2P_{e}P_{AB}^{\mathrm{key}}). \end{array}$$

That is to say, compared with the NRK-SC scheme, our proposed scheme has a higher channel capacity. In addition, Shannon’s second theorem shows that when the information transfer rate is lower than the channel capacity, reliable communication can be guaranteed by channel coding. This means that Bob can recover the same information as Alice.

## 5. Simulation Results

In this section, the performance of our proposed scheme is verified through Monte Carlo simulations. The experiment is repeated $10^{6}$ times in each simulation. We use a single antenna for Alice, Bob, and Eve. The wireless channel is modeled as a Rayleigh fading channel. In addition, the transmitted bits are modulated by BPSK. When simulating the BER of the coding channel and the secure transmission rate, we assume that the power allocation for the pilot and data signals is the same, i.e., α=0.5.

**Figure 6:**
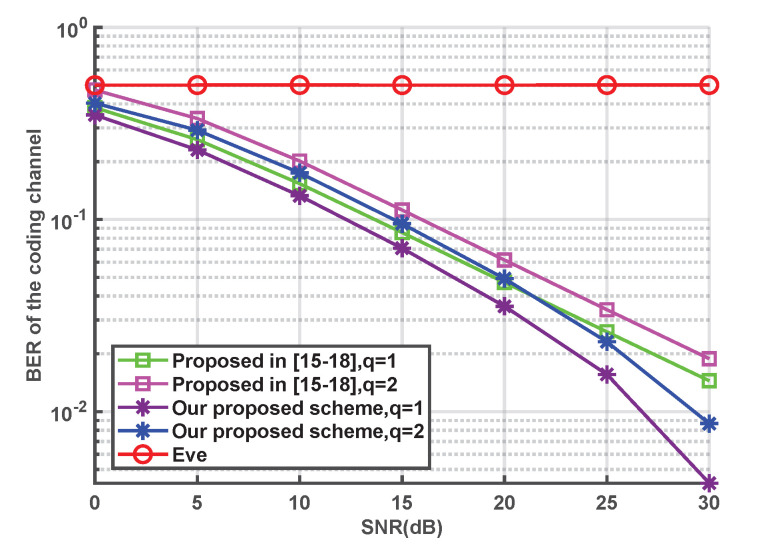
Comparison of the BERs of the coding channels [15,16,17,18].

The BER performance of the coding channel with different quantization precisions *q* in the two schemes is illustrated in Figure 6. It is obvious that the BER of our proposed scheme is lower than that of the NRK-SC scheme in [15,16,17,18] at the same quantization precision, which verifies the correctness of the theoretical derivation. The reason for this is that our proposed scheme makes full use of channel correlation, and there are similar error probabilities between keys and transmitted bits. Thus, the errors that occur in both keys and transmitted bits can be corrected via an XOR operation, which is conducive to reducing the BER of the coding channel. In addition, note that the BER between Alice and Eve is always kept at 0.5 as the SNR increases, which indicates that Eve cannot obtain any private information from the key, regardless of the computing power. This is due to the fact that the generated keys are firmly bound to the channel characteristics. Furthermore, the eavesdropping channel and the legitimate channels are independent of each other. Therefore, the generated keys between Eve and Alice are not correlated.

In order to verify the superiority of our proposed adaptive coding algorithm, a comparison between adaptive coding and the single-code-rate coding method in different channel environments is given in Table 1. We take a low SNR of 0 dB and a high SNR of 20 dB as examples for analysis. We compare the code rate and the BER after decoding using BCH code with code length n=127. When the BER is lower than $10^{-6}$, we consider that reliable transmission can be achieved. That is, the BER is equal to 0 in Table 1. It is observed that the BER using adaptive coding and coding with 1/4 code rate can satisfy the BER requirements at 0 dB and 20 dB. However, it is noted that the code rate of adaptive coding is higher than 1/4, and therefore a higher secure transmission efficiency can be achieved. In addition, when the SNR is 0 dB, The BER using the error correction for the 1/2 code rate is $10^{-4}$, which cannot realize the reliability of communication systems. At 20 dB SNR, although using the 1/2 code rate to correct errors can meet the BER requirements, the code rate is lower than that of adaptive coding. Meanwhile, adding more redundant code bits will lead to lower secure transmission efficiency.

**Figure 7:**
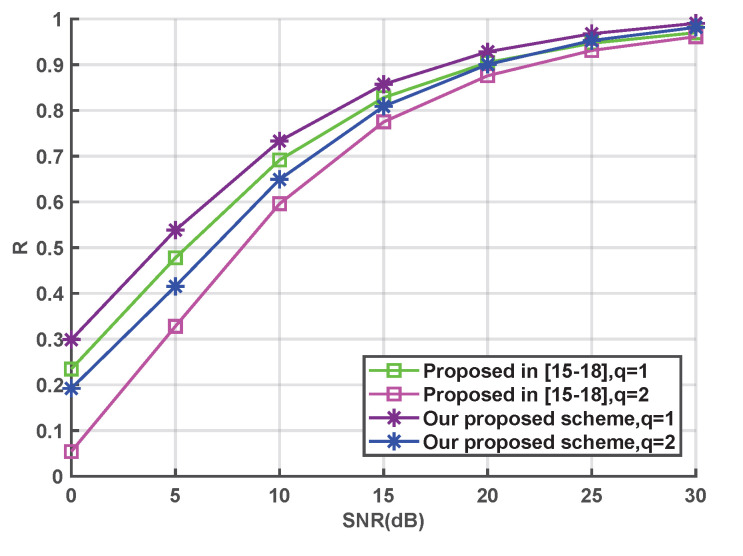
Comparison of secure transmission rates [15,16,17,18].

The upper bound of the secure transmission rate versus SNR is plotted in Figure 7. The secure transmission rate of our proposed scheme is higher than that of the existing NRK-SC scheme in [15,16,17,18] at the same quantization precision. This is because the BER is obviously decreased in our scheme, and fewer redundant code bits are required to correct errors. Therefore, a higher secure transmission rate can be achieved.

**Figure 8:**
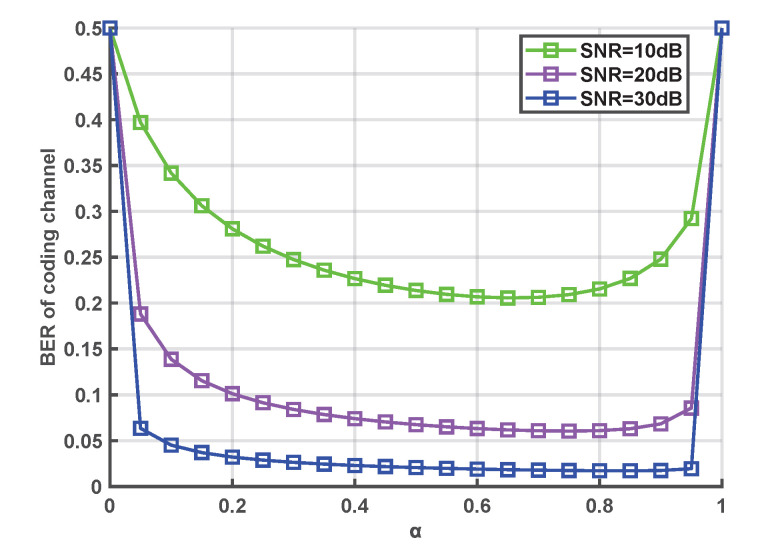
BER comparison for different power allocation parameters.

Reasonable power allocation can minimize the BER of communication systems, which has an important impact on improving the reliability of communication systems, especially in resource-limited communication systems. In order to achieve optimal power allocation, the BER of the coding channel versus the power allocation parameter is given in Figure 8. Note that the minimum values of the BER at different SNRs are obtained at α>0.5. This is due to the fact that the errors of communication systems include inconsistent keys and transmitted error bits, and most of the errors originate from inconsistent keys. Therefore, more power should be appropriately allocated to pilot signals in the design of communication systems. Clearly, when the power allocation parameter is between 0.6 and 0.8, the BER is lower than for other power allocation parameters.

## 6. Conclusions

This paper presented a non-reconciled physical-layer keys-assisted secure communication scheme based on channel correlation. The design of this scheme included three steps, namely, signal frame design, non-reconciled key generation, and secure transmission based on adaptive coding. The proposed signal frame utilizes channel correlation between non-reconciled key generation and secure transmission to reduce the BER of the communication system. Moreover, the proposed adaptive coding algorithm based on the equivalent channel can maximize the secure transmission rate at different SNRs. Theoretical analysis verified the security and reliability of this scheme. Furthermore, simulation results showed a significant performance improvement for this scheme in terms of BER and secure transmission rate.

## Figures and Tables

**Table 1:** Comparison of different coding methods.

SNR (BER, Code Rate) Coding Method	Low Code Rate (1/4)	Adaptive Coding	High Code Rate (1/2)
Low SNR (0 dB)	(0, 1/4)	(0, 0.28)	($10^{-4}$, 1/2)
High SNR (20 dB)	(0, 1/4)	(0, 0.94)	(0, 1/2)

## Data Availability

Not applicable.
